# Culture and Social Change in Mothers’ and Fathers’ Individualism, Collectivism and Parenting Attitudes

**DOI:** 10.3390/socsci10120459

**Published:** 2021-11-30

**Authors:** Jennifer E. Lansford, Susannah Zietz, Suha M. Al-Hassan, Dario Bacchini, Marc H. Bornstein, Lei Chang, Kirby Deater-Deckard, Laura Di Giunta, Kenneth A. Dodge, Sevtap Gurdal, Qin Liu, Qian Long, Paul Oburu, Concetta Pastorelli, Ann T. Skinner, Emma Sorbring, Sombat Tapanya, Laurence Steinberg, Liliana Maria Uribe Tirado, Saengduean Yotanyamaneewong, Liane Peña Alampay

**Affiliations:** 1 Center for Child and Family Policy, Duke University, Durham, NC, USA; 2 Center for Child and Family Policy, Duke University, Durham, NC, USA; 3 Department of Special Education, Hashemite University, Zarqa, Jordan; 4 Department of Humanistic Studies, University of Naples “Federico II,” Naples, Italy; 5 *Eunice Kennedy Shriver* National Institute of Child Health and Human Development, Bethesda, MD, USA; UNICEF, New York, USA; and Institute for Fiscal Studies, London, UK; 6 Department of Psychology, University of Macau, Macau, China; 7 Department of Psychological and Brain Sciences, University of Massachusetts Amherst, USA; 8 Department of Psychology, Università di Roma “La Sapienza,” Rome, Italy; 9 Center for Child and Family Policy, Duke University, Durham, NC, USA; 10 Centre for Child and Youth Studies, University West, Trollhättan, Sweden; 11 Maternal and Child Health, School of Public Health and Management, Chongqing Medical University, China; 12 Global Health Research Center, Duke Kunshan University, China; 13 Department of Psychology, Maseno University, Maseno, Kenya; 14 Department of Psychology, Università di Roma “La Sapienza,” Rome, Italy; 15 Center for Child and Family Policy, Duke University, Durham, NC, USA; 16 Centre for Child and Youth Studies,University West, Trollhättan, Sweden; 17 Peace Culture Foundation, Chiang Mai, Thailand; 18 Department of Psychology, Temple University, Philadelphia, USA; Department of Psychology, King Abdulaziz University, Jeddah, Saudi Arabia; 19 Department of Psychology, Universidad de San Buenaventura, Medellín, Colombia; 20 Department of Psychology, Chiang Mai University, Chiang Mai, Thailand; 21 Department of Psychology, Ateneo de Manila University, Quezon City, Philippines

**Keywords:** authoritarian, collectivism, culture, historical perspective, individualism, international, parenting attitudes, social change

## Abstract

Cultures and families are not static over time but evolve in response to social transformations, such as changing gender roles, urbanization, globalization, and technology uptake. Historically, individualism and collectivism have been widely used heuristics guiding cross-cultural comparisons, yet these orientations may evolve over time, and individuals within cultures and cultures themselves can have both individualist and collectivist orientations. Historical shifts in parents’ attitudes also have occurred within families in several cultures. As a way of understanding mothers’ and fathers’ individualism, collectivism, and parenting attitudes at this point in history, we examined parents in nine countries that varied widely in country-level individualism rankings. Data included mothers’ and fathers’ reports (*N* = 1338 families) at three time points in China, Colombia, Italy, Jordan, Kenya, Philippines, Sweden, Thailand, and the United States. More variance was accounted for by within-culture than between-culture factors for parents’ individualism, collectivism, progressive parenting attitudes, and authoritarian parenting attitudes, which were predicted by a range of sociodemographic factors that were largely similar for mothers and fathers and across cultural groups. Social changes from the 20th to the 21st century may have contributed to some of the similarities between mothers and fathers and across the nine countries.

## 1. Introduction

Historically, the individualist versus collectivist distinction has been one of the main organizing frameworks for understanding cultural differences in family life (Hofstede 1980; Kâğıtçıbaşı 1997; Triandis et al. 1986), and parents in some societies have been considered more “progressive” (in terms of holding democratic attitudes about parent–child relationships and believing that children should have more autonomy in decision making in the family) and less authoritarian than others (e.g., Lansford and Bornstein 2011). However, neither cultures nor families are static over time; instead, cultures and families evolve in response to social transformations, such as changing gender roles, urbanization, globalization, and technology uptake (e.g., Bornstein 2019; Chuang et al. 2018; Lansford et al. 2021). Entire cultural orientations can shift, and changing ecological demands resulting from social transformations can alter parents’ attitudes if they perceive that new parenting behaviors, child characteristics, or both will be more adaptive in altered social contexts (e.g., Fung et al. 2017). The present study examines the proportions of variance in individualism, collectivism, parents’ progressive attitudes, and parents’ authoritarian attitudes accounted for by within-culture versus between-culture factors as well as sociodemographic predictors of individualism, collectivism, parents’ progressive attitudes, and parents’ authoritarian attitudes, recognizing that these constructs are culturally and historically grounded in ways that may change over time.

### 1.1. Individualism and Collectivism in Historical Perspective

Individualism is characterized by self-reliance and separation from ingroups, whereas collectivism is characterized by the subordination of individual goals for the good of the group, interdependence, and family integrity (Triandis et al. 1986). In his now-classic work, Hofstede (1991) explained that “Individualism stands for a society in which the ties between individuals are loose; everyone is expected to look after himself or herself and his or her immediate family only” whereas “collectivism stands for a society in which people from birth onwards are integrated into strong, cohesive ingroups, which throughout people’s lifetime continue to protect them in exchange for unquestioning loyalty” (pp. 260–261). Kâğıtçıbaşı (1997) traced the history of individualism and collectivism in philosophical and religious thought through the first part of the 20th century, but Hofstede (1980) popularized the idea of individualism and collectivism in psychology. The concepts rapidly gained traction in the 1980s and the 1990s, with a third of psychology studies invoking individualism and collectivism to explain cultural differences by 1994 (Hui and Yee 1994). The introduction to Volume 3 of the 2nd edition of *Handbook of Cross-cultural Psychology* noted that, although the 1st edition of the handbook barely mentioned the individualism/collectivism heuristic, the 2nd edition “makes very clear that individualism/collectivism is currently the favorite heuristic of many cross-cultural social psychologists” (Segall and Kâğıtçıbaşı 1997, p. xxvii).

Despite the importance of the individualism/collectivism heuristic, critics have raised a number of questions about these constructs (Voronov and Singer 2002). For example, even in early studies, scholars recognized as a limitation that individualism and collectivism are rarely measured directly but rather assumed based on nationality or ethnicity (Kâğıtçıbaşı 1997). In a comparison of tendencies to give and expectations to receive resources from others in Greece, Hong Kong, the Netherlands, Turkey, and the United States, the presumed collectivists did not differ from the presumed individualists (Fijneman et al. 1995). Indeed, even early theorists pointed out that individualism and collectivism are not polar opposites but instead can coexist within a cultural group and even within an individual in different situations or at different times (Kâğıtçıbaşı 1997).

Over time, researchers have increasingly emphasized that cultural groups as a whole as well as individuals within cultures embody both individualism and collectivism. For example, in an analysis of change over time in individualism and collectivism in Japan, although Japanese culture as a whole was found to become more individualistic over time, individuals continued to embrace many attitudes and behaviors characteristic of collectivism (Ogihara 2017). Some of this shift in perspectives in the literature is likely a function of researchers recognizing complexity that had always existed but had been simplified through dichotomously classifying cultural groups as being predominantly individualist or collectivist (e.g., Wong et al. 2018). Yet some of this shift in perspectives is also likely a function of real societal changes over time related to changing gender roles, urbanization, globalization, technology uptake, and other factors (e.g., Chang et al. 2011; Kâğıtçıbaşı 2002).

In addition to being used as heuristics for understanding cultures in general, individualism and collectivism have been conceptualized as affecting how parents in different cultural groups socialize their children. For example, individualism has been theorized to promote socializing children to be self-reliant and independent, whereas collectivism has been theorized to promote socializing children to be obedient and fulfill their duties to their families (Triandis et al. 1990). Individualism and collectivism may affect parents’ attitudes regarding appropriate parenting practices and desired child outcomes (He et al. 2021).

At a cultural level, individualism and collectivism have typically been treated as characterizing populations to different degrees (e.g., Hofstede Insights 2021). Individualism and collectivism may be related to a range of sociodemographic characteristics at both cultural and individual levels. Early works suggested that more individualistic countries have higher gross national products than less individualistic countries (Hofstede 1980; Triandis et al. 1988), but more recent work demonstrates that individualism and collectivism may have complex relations with economic factors, such as redistribution of income and entrepreneurship, at a societal level (Binder 2019). Culture-wide indicators of educational attainment do not have clear relations with individualism or collectivism, as countries that perform among the best in the world on international comparisons, such as with the Program for International Student Assessment (U.S. Department of Education 2020), include some of the most collectivist (e.g., China, Singapore) and most individualist (e.g., Canada, Estonia) countries in the world. There is some evidence that as countries’ individualism increases over historical time, the average family size decreases (Ogihara 2018). At an individual level, it is also possible that individualism and collectivism are predicted by a range of sociodemographic characteristics of parents, such as age, education, and income. However, research on individual-level predictors of individualism and collectivism is rare.

### 1.2. Progressive and Authoritarian Parenting Attitudes in Historical Perspective

Along with changes in individualism and collectivism at the cultural level, historical shifts in parents’ attitudes and behaviors have also occurred within families (Haring et al. 2019). For example, in previous generations, authoritarian attitudes that emphasized parents’ power and children’s obligation to obey their parents were more common than they are today (Chang et al. 2011; Chen and Chen 2010). Historical shifts in parents’ attitudes in a number of countries have de-emphasized authoritarianism and increasingly emphasized supporting children’s autonomy (Bray and Dawes 2016). Parents’ behaviors likewise have changed. For example, the percentage of parents who report spanking their children (often considered a behavioral manifestation of authoritarianism) has declined steadily over time in a number of countries and has declined more dramatically in countries that have outlawed corporal punishment (Alampay et al. 2021), a number that has increased exponentially since 1979 when Sweden became the first country to outlaw corporal punishment (www.endcorporalpunishment.org, accessed 26 November 2021).

Large-scale social changes are in part responsible for changes in parents’ attitudes (Chang et al. 2011). For example, the Internet and social media have dispersed global perspectives that were not part of traditional family discourse in the era before the Internet (Harrelson-Stephens and Callaway 2014). Parenting has been shaped by exposure to different perspectives via technology, as well as through urbanization, globalization, and other social forces that change over time (Bray and Dawes 2016), and these exposures may have contributed to melding of individualist and collectivist orientations as well as a shift away from more authoritarian parenting attitudes.

Parents’ attitudes are shaped by a number of sociodemographic factors at both a cultural level and an individual level. For example, at a cultural level, some countries emphasize a democratic approach to parenting that encompasses children’s rights in the family and society at large (e.g., Sorbring et al. 2021), whereas other countries emphasize more authoritarian parenting attitudes within the context of hierarchical parent–child relationships (Osman et al. 2021), although these culture-level differences in parenting attitudes may be narrowing over time (Chang et al. 2011). At an individual level, parents who are less educated and have a lower income have more authoritarian attitudes than more educated and higher-income parents (Hoff and Laursen 2019; Wamser-Nanney and Campbell 2020), but studies of these sociodemographic predictors of parents’ attitudes have largely been conducted in the United States, Canada, and western Europe, so it is not clear whether they generalize to other populations. Child gender, parent age, and family size may also be related to parents’ authoritarian attitudes, although findings have been mixed and if differences are found, they are often small in magnitude. The importance parents place on religion has sometimes been found to be related to more authoritarian parenting attitudes (Howarth and Lees 2010), but this finding has been inconsistent (Petro et al. 2018) and in part depends on religious denomination as some denominations espouse more authoritarian beliefs than others (such as the “spare the rod, spoil the child” view in conservative Christian denominations) (Gershoff et al. 1999).

Parents’ own sociodemographic characteristics, in particular age and education, might be related not only to their own attitudes but also to the other parents’ attitudes. Family Systems Theory (Bowen 1978) and expansions of the theory that focus more on culture (Erdem and Safi 2018), for example, describe how families operate as entire systems in which characteristics of each member influence each of the other members, as well as their relationships with one another. In addition, through assortative mating, parents with particular sociodemographic characteristics often select into relationships with partners who share those characteristics (Rauscher 2020). Once mothers and fathers have formed a partnership, they also influence each other over time (Bornstein et al. 2011b), which could account for how one parent’s age and education might be related not only to their own attitudes but also to the other parent’s attitudes.

### 1.3. Mothers and Fathers in Historical Perspective

Historically, in many countries, men were expected to be providers and disciplinarians and women were expected to be children’s primary caregivers (Rodrigo et al. 2014). Gender roles have changed over time as a function of many factors, including women’s attainment of higher education, greater participation in the paid labor force, and increased access to birth control that has given women more control over family planning (Miho and Thévenon 2020). In some countries, paid paternity leave, in addition to maternity leave, has also encouraged fathers to take more active roles in caring for their children (International Labor Organization 2014). As a result, fathers in many countries spend more time with their children and are more involved parents in the 21st century than they were in the 20th century, although fathers’ involvement depends on a number of sociodemographic characteristics, such as parental education (Dotti Sani and Treas 2016).

Changes in parents’ roles over historical time may be tied to changes in parenting attitudes. In particular, fathers’ traditional disciplinarian role may have been related to holding more authoritarian attitudes, but as fathers increasingly take on caregiving roles, they may become less authoritarian. A study in Sweden, for example, demonstrated a dramatic decrease over the last 50 years of authoritarian parenting of both mothers and fathers and increasing egalitarianism between mothers and fathers (Trifan et al. 2014). Likewise, noticeable cross-century change from gender-differentiated authoritarian to gender-equal progressive parenting roles has been documented in China (Chang et al. 2011). Examining both mothers’ and fathers’ individualism, collectivism, and parenting attitudes is important in advancing understanding of both parents, especially as fathers have taken on more active parenting roles over historical time (Craig et al. 2014).

### 1.4. The Present Study

As a way of understanding mothers’ and fathers’ individualism, collectivism, and parenting attitudes at this point in history, we examined parents in nine countries that varied widely in Hofstede Insights’ (2021) individualism rankings: 13 (Colombia), 20 (China and Thailand), 25 (Kenya), 30 (Jordan), 32 (Philippines), 71 (Sweden), 76 (Italy), and 91 (United States). This range enables us to test our research questions in an international sample that varies in individualism at the country level. Hofstede does not provide collectivism rankings, which presumes that individualism and collectivism are reciprocally related.

Our first aim was to understand the proportion of the total variance accounted for by within-culture versus between-culture factors in (1) parental individualism, (2) parental collectivism, (3) parental progressive attitudes, and (4) parental authoritarian attitudes. We hypothesized that a larger proportion of variance would be accounted for by within-culture than between-culture factors, as suggested by prior research on a range of parenting and child development variables (Deater-Deckard et al. 2018) and theories that emphasize that individualism and collectivism can coexist within cultures and within individuals (Kâğıtçıbaşı 1997). Parsing within-culture and between-culture variance in individualism, collectivism, and parenting attitudes is important to understanding the utility of these heuristics as ways of categorizing cultural groups and as a way of understanding the degree to which parents’ orientations and attitudes are predicted by both cultural and individual factors. Our second aim was to understand possible sociodemographic predictors of mothers’ and fathers’ individualism, collectivism, progressive attitudes, and authoritarian attitudes. We hypothesized that parents who are less educated and have lower levels of household income would have less progressive and more authoritarian attitudes than parents who are more educated and have higher levels of household income. We also examined child gender, parent age, number of adults in the household, number of children in the household, and the importance mothers place on religion as predictors. Previous research has been mixed on whether these factors predict parenting attitudes, so we did not make specific directional hypotheses. Examining sociodemographic predictors of individualism, collectivism, and parenting attitudes is important to situate the study of parenting not just in the cultural contexts in which it occurs but also in the individual-level predictors that might be important to understanding culturally grounded orientations and attitudes. Our third aim was to examine whether these predictors significantly differ between mothers and fathers or across cultures. We did not have specific hypotheses about parent gender or cultural differences in predictors but sought to understand generalizability of findings across these dimensions. A key goal in psychological science has become establishing the robustness and replicability of findings (Bonett 2012; Duncan et al. 2014), so testing whether the findings are consistent for mothers and fathers and across cultural groups is important to understanding the generalizability of the findings across diverse populations.

## 2. Methods

### 2.1. Participants

Children, mothers, and fathers (*N* = 1338 families) were recruited to participate in the Parenting Across Cultures Project from schools in 12 groups in 9 countries: The families were recruited from Shanghai, China (*n* = 123); Medellín, Colombia (*n* = 108); Naples, Italy (*n* = 102); Rome, Italy (*n* = 111); Zarqa, Jordan (*n* = 114); Kisumu, Kenya (*n* = 100); Manila, Philippines (*n* = 120); Trollhättan/Vänersborg, Sweden (*n* = 129); Chiang Mai, Thailand (*n* = 120); and Durham, North Carolina, United States (*n* = 102 Black, *n* = 99 Latinx, *n* = 110 White). Children brought home letters describing the study, which parents were asked to sign and return if they were willing to be contacted (in some countries) and contacted by phone to follow up on the letter (in other countries). Children were sampled from schools serving high-, middle-, and low-income families in the approximate proportion to which these income groups were represented in the local population. These sampling procedures resulted in an economically diverse sample that ranged from low income to high income within each site. These are convenience samples, which despite their limitations in terms of population-wide generalizability, have several advantages in longitudinal, developmental research (Jager et al. 2017).

At Time 1 in 2008, children ranged in age from 7 to 10 years (M = 8.30, SD = 0.66; 51% girls). Eighty-two percent of the parents were married. For the analyses reported here, data were available from three annual waves of data collection, spaced at approximately 1-year intervals. At Time 3, 91% of the original families provided data. Compared to the original families who did not provide Time 3 data, families who provided Time 3 data did not differ with respect to demographic variables, including child gender, parents’ marital status, and parents’ education.

### 2.2. Procedure and Measures

Measures were translated and back translated and subjected to a process of cultural adaptation to ensure linguistic and conceptual equivalence of the measures (Erkut 2010). After parents provided informed consent and children provided assent, interviews were conducted face-to-face or over the telephone. Participants were given modest compensation for their time.

Table 1 provides means, standard deviations, and sample sizes for each variable in each site. Table 2 provides the bivariate correlations with the Bonferroni-adjusted significance level across the variables used in the model for all countries combined.

Parent individualism and collectivism: When the children were 10 years old, the mothers and fathers completed a measure of individualism and collectivism adapted from Singelis et al. (1995), Tam et al. (2003), and Triandis (1995). The parents rated the importance of different values related to their autonomy and belonging to a social group. The parents were asked whether they 1 = strongly disagree, 2 = disagree, 3 = agree, or 4 = strongly agree with a series of 16 statements, 8 reflecting individualism and 8 reflecting collectivism. Individualism items included “I’d rather depend on myself than others” and “Competition is the law of nature.” Collectivism items included “The well-being of my co-workers is important to me” and “To me, pleasure is spending time with others.” Items were averaged to create an individualism scale (αs = 0.70 and 0.71 for mothers and fathers, respectively, ranging from 0.52 in Kenya to 0.82 in U.S. Latinx, with 66% over 0.60 for mothers, and from 0.53 in Rome, Italy, to 0.77 in Naples, Italy, with 83% over 0.60 for fathers) and a collectivism scale (αs = 0.65 and 0.69 for mothers and fathers, respectively, ranging from 0.55 in U.S. White to 0.81 in U.S. Latinx, with 83% over 0.60 for mothers, and from 0.59 in Naples, Italy, to 0.80 in China, with 92% over 0.60 for fathers).

Parental progressive and authoritarian attitudes: The Parental Modernity Index (Schaefer and Edgerton 1985) was completed at child age 8. The mothers and fathers rated each of 30 statements on a 4-point scale (1 = strongly disagree, 4 = strongly agree). Of these, 8 items reflect progressive attitudes (e.g., “Children have a right to their own point of view and should be allowed to express it”) and 22 items reflect authoritarian and conformist attitudes (e.g., “The most important thing to teach children is absolute obedience to their parents”). The items were averaged to create a progressive attitudes scale (αs = 0.57 and 0.56 for mothers and fathers, respectively, ranging from 0.37 in Kenya to 0.74 in Thailand, with 42% over 0.60 for mothers, and from 0.40 in Naples, Italy, to 0.70 in Sweden, with 42% over 0.60 for fathers) and an authoritarian attitudes scale (αs = 0.88 and 0.88 for mothers and fathers, respectively, ranging from 0.70 in Jordan to 0.88 in the Philippines for mothers and from 0.70 in Thailand to 0.90 in US Latinx for fathers).

Predictors: At child ages 8 and 9, the mothers completed a demographic questionnaire either orally or in writing (depending on the mothers’ preference) that included items about the number of years of education completed by the mother and the father (in both years) and the household income in local currency (only in year 2). We standardized the education measures and the year 2 household income within site to aid in comparison of structural coefficients, because income and education, even when converted to common units, often do not have comparable meanings between nations and cultural groups. Additional demographic predictors included child gender, mother age, father age, number of adults in the household, and number of children in the household. The mothers were also asked to rate “how important would you say religion is in your life” on a 5-point scale, where 1 = not important and 5 = very important.

### 2.3. Analysis Plan

All continuous variables were standardized to a grand mean of 0 and an SD of 1 to yield easily interpretable relations between predictors and outcomes. We handled missing data using Full Information Maximum Likelihood (Larsen 2011). Data were missing on 13% of the mothers and 28% of the fathers on the individualism/collectivism variables and 5% of the mothers and 23% of the fathers on the parental progressive and authoritarian attitude variables.

For aim 1, the within-culture variance and between-culture variance was calculated using a fixed-effects multilevel model without predictors in Mplus (also known as an unconditional model). The model estimates the within-level and between-level variances, which were used to calculate the intra-class correlation (ICC), which measures the proportion of inter-person variance that is between cultures.

For aims 2 and 3, we estimated the a priori model testing predictors of mothers’ and fathers’ individualism, collectivism, progressive attitudes, and authoritarian attitudes using a structural equation multiple group model by site using Mplus version 8. We used the MLR estimator to provide Satorra–Bentler robust standard errors to address any non-normality in the dependent variables. China was not included in the outcome analysis due to having no data on household income. Because we started with the theoretical perspective that the hypothesized relations should be universal and not differ by culture, we estimated a model that held all paths and correlations of the outcomes to be equal across cultures. A good model fit is defined by a non-significant chi-square test, CFI and TLI greater than or equal to 0.95, and RMSEA less than or equal to 0.06 (Hu and Bentler 1999). If a good model fit was not achieved, modification indices were then consulted to determine parameters that would be theoretically plausible to free in a specific culture. The parameter with the largest index was freed in a specific culture and no longer constrained to be equal across cultures. This model with unconstrained parameters was compared to a fully constrained model using a chi-square difference test. If the test revealed a significant difference in fit, then the unconstrained parameter was retained, modification indices were again consulted, and the plausible parameter with the largest index was freed. This iterative process was continued until the chi-square test comparing the constrained and unconstrained models was not significant, indicating that the model freeing the parameter (less parsimonious model) fit the data significantly worse than the more parsimonious fixed model. We then pruned paths that were non-significant for all cultures. We conducted a sensitivity analysis using more conservative bootstrapped standard errors in place of MLR, and the significance and magnitude of the findings were not different upon comparison with the initial model.

## 3. Results

### 3.1. Within- vs. Between-Culture Variance in Individualism, Collectivism, Progressive Attitudes, and Authoritarian Attitudes

Our first research aim was to understand the proportions of the total variance in parental (1) individualism, (2) collectivism, (3) progressive attitudes, and (4) authoritarian attitudes accounted for by within-culture versus between-culture factors. For each variable, the majority of the variance was between individuals within cultures (Table 3). The ICCs ranged from 0.03 for father collectivism to 0.35 for mother authoritarian attitudes, meaning that 3% of the variance in father collectivism and 35% of the variance in mother authoritarian attitudes were accounted for by between-culture factors. The strongest ICCs were for mother and father individualism (0.22 and 0.19) and mother and father authoritarian attitudes (0.35 and 0.34). Mother and father collectivism and mother and father progressive attitudes had weak and/or non-significant ICCs.

### 3.2. Predictors of Individualism, Collectivism, Progressive Attitudes, and Authoritarian Attitudes

Our second and third research aims concerned potential predictors of parental (1) individualism, (2) collectivism, (3) progressive attitudes, and (4) authoritarian attitudes and whether these relations differed significantly across (1) mothers and fathers and (2) cultures. Bivariate correlations are presented in Table 2.

Table 4 provides the unstandardized results for the final multiple group model for culture. Our initial a priori multiple group model constraining all paths to be equal across cultures had a mixed fit (χ2(1088) = 1612.234, *p* < 0.001, RMSEA = 0.067, 90% CI = (0.060, 0.074), CFI = 0.636, TLI = 0.632). After freeing 7.34% (80) of all possible paths, the model fit significantly better (χ2(923) = 957.236, *p* = 0.211, RMSEA = 0.018, 90% CI = (0.000, 0.033), CFI = 0.976, TLI = 0.972). Child gender and number of adults in the household were not significantly associated with any of the outcomes for any of the cultures and are omitted from Table 4.

Mother individualism: The only variables that were significantly associated with mother individualism were mother education, and only in Kenya, where mother education was positively associated with mother individualism, and the number of children in the household in Naples and Rome, Italy, with more children in the household associated with significantly higher individualism in Naples and significantly lower individualism in Rome.

Father individualism: Father education was significantly negatively associated with father individualism, except in Jordan, where the association was positive. In Kenya, mother education was significantly positively associated with father individualism. Religious importance was positively related to father individualism in the Philippines and negatively related to father individualism in U.S. Black Americans.

Mother collectivism: The importance of religion to mothers was positively associated with mother collectivism in all cultures. Additionally, father education was significantly negatively associated with mother collectivism in all cultures except Sweden, where it was not significantly related. Family income was significantly positively associated with mother collectivism in all cultures except for Naples, Italy, and Kenya, where they were not significantly related. Mother collectivism was also negatively related to mother age and mother education in Sweden, father age in Colombia, and number of children in the household in Kenya.

Father collectivism: Father age was significantly negatively associated with father collectivism in all cultures except Colombia. Additionally, the number of children in the household and the mother’s education were positively associated with the father’s collectivism in Kenya.

Mother progressive attitudes: Mother education was significantly positively associated with mother progressive attitudes in all cultures except for Sweden. Additionally, religious importance was significantly negatively associated with mother progressive attitudes in all cultures. Father age was significantly negatively associated with mother progressive attitudes in Sweden.

Father progressive attitudes: Father education and mother age were significantly positively associated with father progressive attitudes in all cultures. Family income was only significantly positively associated with father progressive attitudes in Jordan and not significantly associated in the other cultures. Additionally, the importance of religion was significantly positively associated with father progressive attitudes in the Philippines and significantly negatively associated with father progressive attitudes in Sweden and in U.S. White Americans.

Mother authoritarian attitudes: Mother education and family income were significantly negatively associated with mother authoritarian attitudes in all cultures, whereas the importance of religion was significantly positively associated with mother authoritarian attitudes in all cultures. Father education was negatively associated with mother authoritarian attitudes only in the Philippines.

Father authoritarian attitudes: Mother education, father education, and family income were significantly negatively associated with father authoritarian attitudes in all cultures, whereas the importance of religion was significantly positively associated with father authoritarian attitudes in all cultures.

## 4. Discussion

This study addressed three aims. Our first aim was to understand the proportions of variance accounted for by within-culture versus between-culture factors in mothers’ and fathers’ individualism, collectivism, progressive attitudes, and authoritarian attitudes. Our hypothesis that more variance in individualism, collectivism, progressive attitudes, and authoritarian attitudes would be accounted for by within-culture than between-culture factors was supported. Our second and third aims were to understand predictors of individualism, collectivism, progressive attitudes, and authoritarian attitudes and whether these predictors are similar for mothers and fathers and across cultures. Overall, few sociodemographic predictors significantly predicted either mothers’ or fathers’ individualism, collectivism, progressive attitudes, and authoritarian attitudes. The most consistent predictors were mothers’ and fathers’ education and mothers’ reports of the importance of religion in their lives. Our hypothesis about less parental education and lower household income being related to more authoritarian parenting attitudes was supported. We found more similarities than differences in predictors across cultures as only 7.34% of paths and correlations among outcomes differed across cultural groups.

It was interesting that between-culture factors accounted for more variance in individualism than collectivism. It is possible that a collectivist orientation taps into aspects of social relationships that are more universally valued across cultures than the aspects of self-reliance that are embodied in individualism so that variance in collectivism is more driven by within-culture factors, such as personality. These findings support conceptualizations of individualism and collectivism as being discrete constructs rather than opposite ends of the same dimension (Kâğıtçıbaşı 1997). Correlations between mothers’ individualism and collectivism and fathers’ individualism and collectivism were modest and positive (0.26 and 0.22, respectively). That is, mothers and fathers who were more individualist were also more, not less, collectivist. Although countries are often ranked or categorized in terms of whether they are more collectivist or individualist, individual parents (as well as countries) can have characteristics that are collectivist in addition to characteristics that are individualist.

Between-culture factors also accounted for more variance in authoritarian than progressive attitudes. Many cultures that historically espoused authoritarian parenting attitudes have become less authoritarian over time (Ulferts 2020). Decreasing authoritarian parenting attitudes have been linked to women’s increased participation in higher education and the labor market, urbanization, globalization, and technology uptake (Chen 2019; Chen and Chen 2010; Greenfield 2009), all of which have shaped social change in the late 20th and early 21st centuries (Chang et al. 2011). An increase in more progressive attitudes, which often endorse children’s agency and right to express their own views, likewise has been associated with social changes over historical time. For example, the Convention on the Rights of the Child, ratified by all nations except the United States, asserts children’s right to participate in decisions that affect their lives and has been used as a catalyst for child protection efforts by many nations from the late 20th to the 21st century (UNICEF 2021). In the present study, mothers and fathers with more authoritarian attitudes had less progressive attitudes but the correlations were modest (−0.18 and −0.15, respectively), suggesting only a loose overlap in these attitudes.

Previous research has treated individualism and collectivism largely as culture- or country-wide variables (e.g., Hofstede 1980), focusing less on individual-level predictors of individualism and collectivism. Although individualism and collectivism remain useful heuristics for categorizing cultural groups and countries (Hofstede 2011; Oyserman et al. 2002), it is also useful to consider individual-level predictors of 21st century parents’ individualism and collectivism. We found no consistent predictors of mothers’ individualism across cultural groups, but fathers who were more educated were less individualistic in 10 of the 11 groups. Mothers’ collectivism was predicted by the placement of more importance on religion in all cultural groups, by lower father education in 10 of the 11 groups, and by higher family income in 8 of the 11 groups. Fathers’ collectivism was predicted by younger father age in 10 of the 11 groups. Other significant predictors were less consistent across cultural groups. These findings suggest that although individualism and collectivism have often been treated as country-level constructs, they can also be understood as being predicted by some individual-level factors.

In comparison to individualism and collectivism, progressive and authoritarian attitudes have more often been treated as individual-level variables that are predicted by factors such as parental education and income (e.g., Park and Lau 2016). Consistent with previous research, we also found that, across cultures, more highly educated mothers and fathers had more progressive and less authoritarian attitudes. A higher family income was also related to mothers’ and fathers’ less authoritarian attitudes but was generally unrelated to mothers’ and fathers’ progressive attitudes. Across the cultural groups included in the present study, we also found that the more importance mothers placed on religion, the less progressive their parenting attitudes were (and the less progressive fathers’ attitudes were in some groups) and the more authoritarian their own and fathers’ parenting attitudes were. These results are consistent with some previous research (Howarth and Lees 2010), although previous findings regarding links between religion and authoritarian parenting attitudes have been mixed (Petro et al. 2018).

### 4.1. Strengths and Limitations

The study’s strengths should be considered in light of the limitations. First, although changes in collectivism versus individualism and progressive versus traditional parenting attitudes have been documented in the literature, we were not able to track these changes historically in our own data. Second, the samples in each site were locally representative of the cities from which they were drawn; they are not nationally representative. Findings should not be generalized to entire nations or to cultural groups beyond those studied here. Future research will benefit from studying within-country variation (e.g., by religious group or rural versus urban residence) as well as the between-country variations examined in the present study. Third, the present study focused primarily on sociodemographic predictors of individualism, collectivism, progressive attitudes, and authoritarian attitudes. Future research will benefit from investigating other predictors, such as political leaning, parents’ experiences when they were children, or personality factors, as ways of understanding how these orientations and attitudes develop (Bornstein et al. 2011a). Fourth, we did not look at country-level variables, such as income inequality or homogeneous vs. heterogeneous ethnic groups or religious populations that may be associated with these outcomes. In addition to examining country-level predictors, future research should also examine individualism, collectivism, progressive attitudes, and authoritarian attitudes as predictors of parenting behaviors in diverse cultural groups to better understand 21st century parenting in the context of ongoing social change.

### 4.2. Implications

The findings have several implications for understanding parenting in the 21st century. As parents have access to a diverse range of global perspectives through the Internet and social media and as they have experienced social changes associated with urbanization and globalization, 21st century parents may be even more likely than those in previous generations to have characteristics of both individualism and collectivism (see Tamis-LeMonda et al. 2008). For example, differences in individuals’ individualist and collectivist orientations are playing out in response to the global COVID-19 pandemic. Individuals who have more individualistic orientations are more motivated to be vaccinated after hearing messages about the individual benefits of the vaccine, whereas individuals who have more collectivistic orientations are more motivated to be vaccinated after hearing messages about benefits for their community (Yuan and Chu 2021). Better understanding of predictors of these orientations offers the possibility of targeting behavioral change messages in ways that might address individuals’ concerns and motivations. For example, because collectivism is predicted by placing more importance on religion, public health experts might be encouraged to work through faith communities to explain how virus mitigation measures, such as vaccinations, work for the greater good.

In addition, as parents in the 21st century continue to navigate sometimes rapid social changes, they may find themselves embracing a mix of both progressive and authoritarian attitudes. Sometimes, this mix of progressive and authoritarian attitudes is especially pronounced for families that immigrate from one country to another country that has different historic and current attitudes and can lead to negotiations between parents and children related to discrepant attitudes (e.g., Osman et al. 2021). Because sociodemographic factors at both a cultural level, as found in previous research (Sorbring et al. 2021), and at an individual level, as found in the present study and previous research (Wamser-Nanney and Campbell 2020), predict parents’ attitudes, understanding the attitudes of 21st century parents may be bolstered by understanding historical changes in demographic factors. For example, as the average education levels attained in a population increase over historical time, as has occurred in many countries (UNICEF 2019), or as a population decreases in its religiosity over historical time, as also has occurred in many countries (Ruck et al. 2018), parents’ attitudes may shift as well.

## 5. Conclusions

Taken together, the findings suggest two main conclusions. First, differences in individualism, collectivism, progressive parenting attitudes, and authoritarian parenting attitudes are accounted for more by within-culture than between-culture factors. Second, in ways that are largely consistent for mothers and fathers and across cultures, individualism, collectivism, progressive parenting attitudes, and authoritarian parenting attitudes are predicted by a range of sociodemographic factors, especially mothers’ and fathers’ education and mothers’ reports of the importance of religion in their lives. Changing gender roles, urbanization, globalization, and technology uptake from the 20th to the 21st century may have contributed to some of the similarities between mothers and fathers and across the nine countries included in this study. As parents are affected by social contexts and also influence social change over time, understanding 21st century parenting is dependent on understanding cultural and sociohistorical contexts in which parents are embedded.

## Figures and Tables

**Table 1.** Mean (*SD*) or % by culture.

	China	Colombia	Italy, Naples	Italy, Rome	Jordan	Kenya	Philippines	Sweden	Thailand	U.S.-Black	U.S.-White	U.S.-Latinx
	(*n* = 123)	(*n* = 108)	(*n* = 102)	(*n* = 111)	(*n* = 114)	(*n* = 100)	(*n* = 120)	(*n* = 129)	(*n* = 120)	(*n* = 102)	(*n* = 110)	(*n* = 99)
**Female**	50.4%	52.0%	47.7%	60.0%	49.2%	49.2%	48.8%	52.0%	41.8%	52.5%	55.6%	47.4%
**Mother Age**	35.420 (3.243)	38.140 (5.621)	40.240 (5.089)	32.450 (6.212)	37.958 (6.194)	37.581 (6.179)	38.080 (4.844)	36.902 (8.413)	40.955 (6.332)	32.863 (5.594)	37.028 (7.799)	36.429 (6.033)
**Father Age**	37.983 (3.886)	41.175 (5.673)	43.521 (5.252)	39.280 (6.867)	40.206 (7.087)	39.955 (7.276)	40.465 (5.710)	38.836 (8.016)	42.212 (5.807)	35.096 (7.048)	40.750 (8.781)	41.768 (5.502)
**Mother Education**	13.551 (2.878)	10.140 (4.346)	14.139 (4.067)	10.690 (3.653)	13.608 (4.065)	12.302 (4.761)	13.890 (2.478)	13.647 (2.357)	16.955 (2.843)	9.832 (4.081)	10.639 (5.598)	13.126 (2.179)
**Father Education**	14.000 (3.066)	10.732 (4.165)	13.753 (4.093)	12.290 (3.605)	13.897 (3.839)	12.761 (4.218)	13.682 (2.957)	13.455 (2.658)	17.286 (3.043)	9.614 (3.904)	9.907 (5.316)	13.241 (3.159)
**Household Income**	N/A	3.439 (1.995)	5.069 (2.242)	1.387 (1.032)	4.924 (2.970)	4.140 (1.991)	7.750 (2.103)	5.011 (2.447)	8.566 (2.046)	4.081 (1.893)	3.693 (2.979)	2.254 (1.046)
**Religious Importance**	1.933 (1.140)	4.309 (0.916)	3.284 (1.492)	4.720 (0.621)	4.675 (0.780)	4.548 (0.728)	1.798 (1.040)	4.535 (0.812)	3.648 (1.342)	4.462 (0.951)	4.565 (0.727)	4.811 (0.458)
**Number of Adults in Household**	2.840 (1.058)	2.300 (0.689)	2.068 (0.661)	2.950 (1.381)	3.733 (2.065)	3.328 (1.508)	1.950 (0.753)	1.755 (0.667)	1.973 (0.515)	2.448 (1.045)	2.537 (1.234)	2.582 (1.214)
**Number of Children in Household**	1.252 (0.491)	2.020 (0.738)	1.951 (0.809)	3.680 (1.657)	2.775 (1.363)	1.798 (0.839)	2.228 (0.773)	2.412 (1.285)	2.367 (1.042)	2.711 (1.207)	2.139 (1.139)	3.460 (1.530)
**Mother Individualism**	2.923 (0.388)	2.736 (0.440)	2.564 (0.326)	2.691 (0.468)	2.900 (0.415)	2.614 (0.345)	2.237 (0.352)	2.652 (0.433)	2.482 (0.339)	2.802 (0.528)	2.668 (0.362)	3.105 (0.372)
**Father Individualism**	2.930 (0.380)	2.795 (0.454)	2.621 (0.322)	2.822 (0.517)	2.907 (0.408)	2.698 (0.350)	2.313 (0.341)	2.703 (0.354)	2.615 (0.350)	2.788 (0.417)	2.666 (0.344)	3.151 (0.446)
**Mother Collectivism**	3.182 (0.345)	3.286 (0.318)	3.169 (0.285)	3.182 (0.425)	3.421 (0.335)	3.304 (0.322)	3.247 (0.309)	3.364 (0.335)	3.266 (0.292)	3.450 (0.377)	3.438 (0.294)	3.241 (0.336)
**Father Collectivism**	3.214 (0.361)	3.283 (0.302)	3.151 (0.319)	3.177 (0.442)	3.342 (0.330)	3.312 (0.359)	3.189 (0.338)	3.391 (0.316)	3.237 (0.304)	3.369 (0.379)	3.282 (0.325)	3.242 (0.385)
**Mother Progressive Attitudes**	3.276 (0.332)	3.107 (0.302)	3.155 (0.274)	2.761 (0.388)	3.103 (0.336)	3.214 (0.361)	3.267 (0.256)	3.065 (0.335)	3.220 (0.287)	3.119 (0.356)	3.145 (0.298)	3.209 (0.387)
**Father Progressive Attitudes**	3.156 (0.320)	3.032 (0.312)	3.109 (0.289)	2.780 (0.372)	3.014 (0.320)	3.212 (0.326)	3.271 (0.346)	2.958 (0.293)	3.154 (0.309)	2.999 (0.318)	3.096 (0.326)	3.155 (0.354)
**Mother Authoritarian Attitudes**	2.405 (0.374)	2.892 (0.394)	2.578 (0.381)	3.023 (0.379)	2.926 (0.446)	2.701 (0.377)	2.268 (0.328)	2.729 (0.424)	2.136 (0.370)	3.049 (0.382)	2.795 (0.343)	2.705 (0.305)
**Father Authoritarian Attitudes**	2.390 (0.335)	2.827 (0.435)	2.526 (0.348)	3.016 (0.383)	2.987 (0.407)	2.732 (0.267)	2.321 (0.309)	2.766 (0.377)	2.212 (0.353)	3.008 (0.438)	2.939 (0.412)	2.722 (0.345)

**Table 2.** Means, standard deviations, and correlations for the entire sample.

Variable	*M*	*SD*	1	2	3	4	5	6	7	8	9	10	11	12	13	14	15
1. Mother Age	37.04	6.51															
2. Father Age	40.19	6.75	0.71 **														
3. Mother Education	12.67	4.13	0.22 **	0.13 **													
4. Father Education	12.85	4.13	0.22 **	0.13 **	0.72 **												
5. Household Income	4.55	2.94	0.29 **	0.15 **	0.59 **	0.53 **											
6. Religious Importance	3.93	1.41	−0.00	0.05	−0.06 *	−0.04	−0.03										
7. Number of Adults	2.57	1.30	0.01	0.01	0.01	0.03	0.08 **	0.11 **									
8. Number of Children	2.39	1.30	−0.15 **	−0.02	−0.10 **	−0.07 *	−0.05	0.26 **	0.09 **								
9. Mother Individualism	2.70	0.45	−0.10 **	−0.04	−0.07 *	−0.07 *	−0.09 **	0.15 **	0.09 **	0.11 **							
10. Father Individualism	2.77	0.45	−0.07 *	−0.01	−0.01	−0.07 *	−0.08 *	0.15 **	0.09 **	0.10 **	0.35 **						
11. Mother Collectivism	3.29	0.34	0.05	0.02	0.02	0.00	0.07 *	0.17 **	0.04	0.01	0.26 **	0.03					
12. Father Collectivism	3.26	0.36	0.06	0.00	0.07 *	0.06	0.05	0.11 **	0.05	0.05	0.09 **	0.22 **	0.27 **				
13. Mother Progressive	3.14	0.35	0.11 **	0.06	0.20 **	0.16 **	0.14 **	−0.17 **	−0.07 *	−0.12 **	0.04	−0.01	0.16 **	0.09 **			
14. Father Progressive	3.08	0.35	0.14 **	0.05	0.14 **	0.14 **	0.08 *	−0.15 **	−0.06 *	−0.09 **	−0.02	−0.02	0.05	0.19 **	0.34 **		
15. Mother Authoritarian	2.68	0.47	−0.19 **	−0.11 **	−0.33 **	−0.24 **	−0.26 **	0.40 **	0.11 **	0.21 **	0.28 **	0.14 **	0.10 **	0.04	−0.18 **	−0.24 **	
16. Father Authoritarian	2.71	0.45	−0.17 **	−0.06	−0.28 **	−0.31 **	−0.30 **	0.41 **	0.08 *	0.20 **	0.19 **	0.25 **	0.09 **	0.09 **	−0.21 **	−0.15 **	0.60 **

*Note. M* and *SD* are used to represent the mean and the standard deviation, respectively.

* *p* < 0.05.

** *p* < 0.01.

**Table 3.** Estimated variances and intra-class correlations.

	Variances (SE)		ICC
	Between Adolescents within Cultures	Between Cultures	
Mother Individualism	0.21 (0.02) ***	0.05 (0.02) *	0.22 (0.08) **
Father Individualism	0.20 (0.02) ***	0.04 (0.02) *	0.19 (0.08) *
Mother Collectivism	0.19 (0.01) ***	0.01 (0.00) ***	0.07 (0.02) ***
Father Collectivism	0.13 (0.01) ***	0.00 (0.00) *	0.03 (0.01) *
Mother Progressive Attitudes	0.12 (0.02) ***	0.02 (0.01)	0.13 (0.07)
Father Progressive Attitudes	0.12 (0.01) ***	0.015 (0.007) *	0.12 (0.049) *
Mother Authoritarian Attitudes	0.22 (0.03) ***	0.08 (0.03) **	0.35 (0.08) ***
Father Authoritarian Attitudes	0.20 (0.02) ***	0.07 (0.02) ***	0.34 (0.07) ***

* *p* < 0.05.

** *p* < 0.01.

*** *p* < 0.001.

**Table 4.** Unstandardized results for the culture multiple group model.

	Colombia	Italy, Naples	Italy, Rome	Jordan	Kenya	Philippines	Sweden	Thailand	U.S.-Black	U.S.-White	U.S.-Latinx
Mother Individualism											
Mother Education	−0.03 (0.02)	−0.03 (0.02)	−0.03 (0.02)	−0.03 (0.02)	0.11 (0.05) *	−0.03 (0.02)	−0.03 (0.02)	−0.03 (0.02)	−0.03 (0.02)	−0.03 (0.02)	−0.03 (0.02)
Number of Children	0.01 (0.01)	0.10 (0.04) *	−0.07 (0.03) *	0.01 (0.01)	0.01 (0.01)	0.01 (0.01)	0.01 (0.01)	0.01 (0.01)	0.01 (0.01)	0.01 (0.01)	0.01 (0.01)
**Father Individualism**											
Mother Education	0.01 (0.02)	0.01 (0.02)	0.01 (0.02)	0.01 (0.02)	0.13 (0.05) **	0.01 (0.02)	0.01 (0.02)	0.01 (0.02)	0.01 (0.02)	0.01 (0.02)	0.01 (0.02)
Father Education	−0.06 (0.02) **	−0.06 (0.02) **	−0.06 (0.02) **	0.07 (0.04) *	−0.06 (0.02) **	−0.06 (0.02) **	−0.06 (0.02) **	−0.06 (0.02) **	−0.06 (0.02) **	−0.06 (0.02) **	−0.06 (0.02) **
Religious Importance	−0.00 (0.01)	−0.00 (0.01)	−0.00 (0.01)	−0.00 (0.01)	−0.00 (0.01)	0.10 (0.04) *	−0.00 (0.01)	−0.00 (0.01)	−0.07 (0.04) *	−0.00 (0.01)	−0.00 (0.01)
**Mother Collectivism**											
Mother Age	0.03 (0.01)	0.03 (0.01)	0.03 (0.01)	0.03 (0.01)	0.03 (0.01)	0.03 (0.01)	−0.06 (0.03) *	−0.06 (0.03)	0.03 (0.01)	0.03 (0.01)	0.03 (0.01)
Father Age	−0.07 (0.03) *	0.01 (0.01)	0.01 (0.01)	0.01 (0.01)	0.01 (0.01)	0.01 (0.01)	0.01 (0.01)	0.01 (0.01)	0.01 (0.01)	0.01 (0.01)	0.01 (0.01)
Mother Education	0.01 (0.01)	0.01 (0.01)	0.01 (0.01)	0.01 (0.01)	0.01 (0.01)	0.01 (0.01)	−0.08 (0.03) **	0.01 (0.01)	0.01 (0.01)	0.01 (0.01)	0.01 (0.01)
Father Education	−0.03 (0.01) *	−0.03 (0.01) *	−0.03 (0.01) *	−0.03 (0.01) *	−0.03 (0.01) *	−0.03 (0.01) *	0.04 (0.02)	−0.03 (0.01) *	−0.03 (0.01) *	−0.03 (0.01) *	−0.03 (0.01) *
Family Income	0.06 (0.01) ***	−0.02 (0.03)	0.06 (0.01) ***	0.06 (0.01) ***	−0.07 (0.05)	0.06 (0.01) ***	0.06 (0.01) ***	0.06 (0.01) ***	0.06 (0.01) ***	−0.04 (0.03)	0.06 (0.01) ***
Religious Importance	0.03 (0.01) **	0.03 (0.01) **	0.03 (0.01) **	0.03 (0.01) **	0.03 (0.01) **	0.03 (0.01) **	0.03 (0.01) **	0.10 (0.02) ***	0.03 (0.01) **	0.03 (0.01) **	0.03 (0.01) **
Number of Children	0.01 (0.01)	0.01 (0.01)	0.01 (0.01)	0.01 (0.01)	−0.08 (0.04) *	0.01 (0.01)	0.01 (0.01)	0.01 (0.01)	0.01 (0.01)	0.01 (0.01)	0.01 (0.01)
**Father Collectivism**											
Father Age	0.05 (0.03)	−0.04 (0.02) *	−0.04 (0.02) *	−0.04 (0.02) *	−0.04 (0.02) *	−0.04 (0.02) *	−0.04 (0.02) *	−0.04 (0.02) *	−0.04 (0.02) *	−0.04 (0.02) *	−0.04 (0.02) *
Mother Education	−0.00 (0.01)	−0.00 (0.01)	−0.00 (0.01)	−0.00 (0.01)	0.14 (0.04) **	−0.00 (0.01)	−0.00 (0.01)	−0.00 (0.01)	−0.00 (0.01)	−0.00 (0.01)	−0.00 (0.01)
Number of Children	0.01 (0.01)	0.01 (0.01)	0.01 (0.01)	0.01 (0.01)	0.11 (0.04) *	0.01 (0.01)	0.01 (0.01)	0.01 (0.01)	0.01 (0.01)	0.01 (0.01)	0.01 (0.01)
**Mother Progressive Attitudes**											
Father Age	0.02 (0.01)	0.02 (0.01)	0.02 (0.01)	0.02 (0.01)	0.02 (0.01)	0.02 (0.01)	−0.05 (0.02) *	0.02 (0.01)	0.02 (0.01)	0.02 (0.01)	0.02 (0.01)
Mother Education	0.04 (0.01) **	0.04 (0.01) **	0.04 (0.01) **	0.04 (0.01) **	0.12 (0.03) ***	0.04 (0.01) **	0.00 (0.03)	0.14 (0.03) ***	0.04 (0.01) **	0.04 (0.01) **	0.04 (0.01) **
Religious Importance	−0.02 (0.01) *	0.06 (0.03) *	−0.02 (0.01) *	−0.02 (0.01) *	−0.02 (0.01) *	−0.02 (0.01) *	−0.02 (0.01) *	−0.02 (0.01) *	−0.02 (0.01) *	−0.02 (0.01) *	−0.02 (0.01) *
**Father Progressive Attitudes**											
Mother Age	0.03 (0.01) *	0.03 (0.01) *	0.03 (0.01) *	0.03 (0.01) *	0.03 (0.01) *	0.03 (0.01) *	−0.05 (0.04)	0.03 (0.01) *	0.03 (0.01) *	0.03 (0.01) *	0.03 (0.01) *
Father Education	0.03 (0.01) *	0.03 (0.01) *	0.03 (0.01) *	−0.12 (0.04) **	0.03 (0.01) *	0.03 (0.01) *	0.03 (0.01) *	0.14 (0.03) ***	0.03 (0.01) *	0.03 (0.01) *	0.03 (0.01) *
Family Income	0.00 (0.01)	0.00 (0.01)	0.00 (0.01)	0.09 (0.04) *	0.00 (0.01)	0.00 (0.01)	0.00 (0.01)	0.00 (0.01)	0.00 (0.01)	0.00 (0.01)	0.00 (0.01)
Religious Importance	−0.00 (0.01)	−0.00 (0.01)	−0.00 (0.01)	−0.00 (0.01)	−0.00 (0.01)	0.06 (0.03) *	−0.06 (0.02) **	−0.00 (0.01)	−0.00 (0.01)	−0.10 (0.03) **	−0.00 (0.01)
**Mother Authoritarian Attitudes**											
Mother Education	−0.11 (0.01) ***	−0.11 (0.01) ***	−0.11 (0.01) ***	−0.11 (0.01) ***	−0.11 (0.01) ***	−0.11 (0.01) ***	−0.11 (0.01) ***	−0.11 (0.01) ***	−0.11 (0.01) ***	−0.11 (0.01) ***	−0.11 (0.01) ***
Father Education	−0.01 (0.01)	−0.01 (0.01)	−0.01 (0.01)	−0.01 (0.01)	−0.01 (0.01)	−0.11 (0.04) **	−0.01 (0.01)	−0.01 (0.01)	−0.01 (0.01)	−0.01 (0.01)	−0.01 (0.01)
Family Income	−0.03 (0.01) *	−0.03 (0.01) *	−0.03 (0.01) *	−0.03 (0.01) *	−0.03 (0.01) *	−0.10 (0.04) *	−0.03 (0.01) *	−0.12 (0.03) ***	−0.03 (0.01) *	−0.03 (0.01) *	−0.03 (0.01) *
Religious Importance	0.05 (0.01) ***	0.05 (0.01) ***	0.05 (0.01) ***	0.05 (0.01) ***	−0.04 (0.02) *	0.05 (0.01) ***	0.05 (0.01) ***	0.05 (0.01) ***	0.05 (0.01) ***	0.05 (0.01) ***	0.05 (0.01) ***
**Father Authoritarian Attitudes**											
Mother Education	−0.04 (0.01) **	−0.04 (0.01) **	−0.04 (0.01) **	−0.04 (0.01) **	−0.04 (0.01) **	−0.04 (0.01) **	−0.04 (0.01) **	−0.04 (0.01) **	−0.04 (0.01) **	−0.04 (0.01) **	−0.04 (0.01) **
Father Education	−0.10 (0.01) ***	−0.10 (0.01) ***	−0.10 (0.01) ***	−0.10 (0.01) ***	−0.10 (0.01) ***	−0.10 (0.01) ***	−0.10 (0.01) ***	−0.10 (0.01) ***	−0.10 (0.01) ***	−0.10 (0.01) ***	−0.10 (0.01) ***
Family Income	−0.03 (0.01) *	−0.03 (0.01) *	−0.03 (0.01) *	−0.03 (0.01) *	−0.03 (0.01) *	−0.10 (0.04) *	−0.03 (0.01) *	−0.03 (0.01) *	−0.16 (0.04) ***	−0.03 (0.01) *	−0.03 (0.01) *
Religious Importance	0.04 (0.01) **	0.04 (0.01) **	0.04 (0.01) **	0.04 (0.01) **	0.04 (0.01) **	0.04 (0.01) **	0.04 (0.01) **	0.04 (0.01) **	0.04 (0.01) **	0.04 (0.01) **	0.04 (0.01) **

*Note*. Child gender, mother age, father age, religious importance, number of adults in the household, number of children in the household, mother education, father education, and family income were included as predictors in all models. Only predictors that were significant in one or more groups are included in the table.

* *p* < 0.05.

** *p* < 0.01.

*** *p* < 0.001.

## Data Availability

The data presented in this study are available on request from the corresponding author. The data are not publicly available due to privacy and ethics restrictions.
